# Advances in Surface-Enhanced Raman Spectroscopy for Urinary Metabolite Analysis: Exploiting Noble Metal Nanohybrids

**DOI:** 10.3390/bios14120564

**Published:** 2024-11-21

**Authors:** Ningbin Zhao, Peizheng Shi, Zengxian Wang, Zhuang Sun, Kaiqiang Sun, Chen Ye, Li Fu, Cheng-Te Lin

**Affiliations:** 1 Faculty of Electrical Engineering and Computer Science, Ningbo University, Ningbo 315211, China; zhaoningbin@nimte.ac.cn; 2 Qianwan Institute, Ningbo Institute of Materials Technology and Engineering (NIMTE), Chinese Academy of Sciences, Ningbo 315201, China; shipeizheng@nimte.ac.cn (P.S.); sunzhuang@nimte.ac.cn (Z.S.); sunkaiqiang@nimte.ac.cn (K.S.); yechen@nimte.ac.cn (C.Y.); 3 Taiyuan Municipal Construction Group Co., Ltd., Taiyuan 030002, China; wangzengxian_tysz@163.com; 4 Key Laboratory of Marine Materials and Related Technologies, Zhejiang Key Laboratory of Marine Materials and Protective Technologies, Ningbo Institute of Materials Technology and Engineering (NIMTE), Chinese Academy of Sciences, Ningbo 315201, China; 5 Center of Materials Science and Optoelectronics Engineering, University of Chinese Academy of Sciences, Beijing 100049, China; 6 College of Materials and Environmental Engineering, Hangzhou Dianzi University, Hangzhou 310018, China; fuli@hdu.edu.cn

**Keywords:** biomarker detection, plasmonic nanostructures, point-of-care diagnostics, spectroscopic sensing, molecular fingerprinting

## Abstract

This review examines recent advances in surface-enhanced Raman spectroscopy (SERS) for urinary metabolite analysis, focusing on the development and application of noble metal nanohybrids. We explore the diverse range of hybrid materials, including carbon-based, metal–organic-framework (MOF), silicon-based, semiconductor, and polymer-based systems, which have significantly improved SERS performance for detecting key urinary biomarkers. The principles underlying SERS enhancement in these nanohybrids are discussed, elucidating both electromagnetic and chemical enhancement mechanisms. We analyze various fabrication methods that enable precise control over nanostructure morphology, composition, and surface chemistry. The review critically evaluates the analytical performance of different hybrid systems for detecting specific urinary metabolites, considering factors such as sensitivity, selectivity, and stability. We address the analytical challenges associated with SERS-based urinary metabolite analysis, including sample preparation, matrix effects, and data interpretation. Innovative solutions, such as the integration of SERS with microfluidic devices and the application of machine learning algorithms for spectral analysis, are highlighted. The potential of these advanced SERS platforms for point-of-care diagnostics and personalized medicine is discussed, along with future perspectives on wearable SERS sensors and multi-modal analysis techniques. This comprehensive overview provides insights into the current state and future directions of SERS technology for urinary metabolite detection, emphasizing its potential to revolutionize non-invasive health monitoring and disease diagnosis.

## 1. Introduction

Surface-enhanced Raman spectroscopy (SERS) has emerged as a powerful analytical technique in recent years, offering unprecedented sensitivity and specificity for molecular detection. This spectroscopic method exploits the phenomenon of plasmon resonance in metallic nanostructures to dramatically amplify Raman signals, enabling the detection of molecules at extremely low concentrations, even down to the single-molecule level [1,2]. The ability of SERS to provide rich structural information about target analytes, coupled with its non-destructive nature, has positioned it as a valuable tool across various scientific disciplines, including chemistry, biology, and medical diagnostics. In the realm of bioanalysis, SERS has shown particular promise for the detection and quantification of metabolites in biological fluids [3,4]. At the same time, with the advancement of artificial intelligence, a growing number of computational models have emerged. When combined with SERS detection methods, these models can transform the complex information contained in the spectra of biological fluids into more accurate and practical diagnoses [5]. Among these, urine stands out as an especially attractive medium for metabolite analysis due to its non-invasive collection process and its rich composition of metabolic end-products that reflect various physiological processes within the body [6]. Urinary metabolite analysis has long been recognized as a valuable approach for assessing overall health status, diagnosing diseases, monitoring treatment efficacy, and evaluating environmental exposures [7]. Traditional methods for urinary metabolite analysis, such as chromatography and mass spectrometry, while highly sensitive and specific, often require extensive sample preparation, are time-consuming, and necessitate sophisticated instrumentation [8]. The electrochemical analysis method exhibits high sensitivity; however, the complex modification of electrodes is prone to contamination, which can lead to interference [9,10]. The fluorescence colorimetric method is indeed an excellent analytical method. But its fluorescent properties are not applicable to all substances present in various urinary environments [11]. The colorimetric method offers distinct advantages over others for urine detection due to its simple operation, rapid detection, minimal equipment requirements, wide range of applications, and the ability for visual quantification. However, there are issues such as severe interference and low sensitivity [12]. SERS, on the other hand, offers the potential for the rapid, sensitive, and potentially portable analysis of urinary metabolites with minimal sample preparation [13,14].

The key to unlocking the full potential of SERS for urinary metabolite analysis lies in the development of advanced SERS substrates. Noble metal nanoparticles, particularly those of gold and silver, have been the cornerstone of SERS substrates due to their strong plasmonic properties [15,16]. However, the field has recently witnessed a paradigm shift toward the development of hybrid nanomaterials that combine noble metals with other functional materials. These composite materials, formed by the combination of noble metal nanoparticles with other materials (such as semiconductors, polymers, carbon-based materials, etc.), are collectively referred to as noble metal nanohybrids. These noble metal nanohybrids offer several advantages over traditional noble metal nanoparticles, including enhanced stability, improved reproducibility, and the potential for multifunctionality [17,18]. This review aimed to provide a comprehensive overview of the recent advances in the application of SERS, specifically focusing on noble metal nanohybrids, for the analysis of urinary metabolites. We explored the diverse range of hybrid nanomaterials that have been developed for this purpose, including carbon-based hybrids, metal–organic-framework (MOF) hybrids, silicon-based structures, semiconductor hybrids, and polymer-based systems. Each of these hybrid materials brings unique properties to the table, offering opportunities to tailor SERS substrates for specific analytical challenges in urinary metabolite detection. The principles underlying SERS enhancement in noble metal nanohybrids were discussed in detail, elucidating both the electromagnetic and chemical enhancement mechanisms. Understanding these principles is crucial for the rational design of hybrid SERS substrates with optimized performance. We then delved into the fabrication methods for these hybrid nanomaterials, highlighting innovative approaches that enable precise control over nanostructure morphology, composition, and surface chemistry [19,20].

A significant portion of this review is dedicated to examining the application of noble metal nanohybrids for the SERS detection of specific urinary metabolites. We focused on key metabolites such as uric acid, urea, creatinine, and dopamine, which serve as important biomarkers for various physiological and pathological conditions. The analytical performance of different hybrid systems for these metabolites was critically evaluated, considering factors such as sensitivity, selectivity, reproducibility, and stability. Additionally, we explored the detection of other relevant urinary components, including proteins and pathogenic bacteria, showcasing the versatility of SERS-based approaches.

The review also addressed the analytical challenges associated with SERS-based urinary metabolite analysis. These include issues related to sample preparation, matrix effects, quantification strategies, and data analysis. We discussed innovative solutions that have been developed to overcome these challenges, such as the integration of SERS with microfluidic devices for improved sample handling and the application of machine learning algorithms for spectral interpretation.

## 2. Principles of SERS and Noble Metal Nanohybrids

SERS has revolutionized molecular detection capabilities, offering unprecedented sensitivity and specificity. This powerful analytical technique harnesses the unique optical properties of noble metal nanostructures to dramatically amplify Raman signals from nearby molecules. To fully appreciate the potential of SERS for urinary metabolite analysis, it is essential to understand the fundamental principles underlying this phenomenon and the role of noble metal nanohybrids in enhancing its effectiveness.

### 2.1. Mechanisms of SERS Enhancement

**Figure 1:**
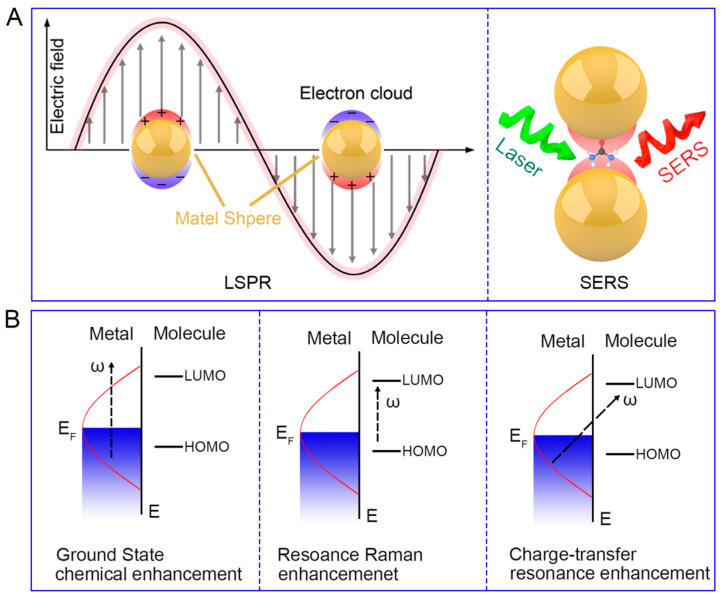
Schematic diagram of EM (**A**) and CM (**B**).

The SERS effect arises from two primary mechanisms: electromagnetic enhancement (EM) and chemical enhancement (CM). The electromagnetic enhancement mechanism, which contributes the majority of the signal amplification, is based on the excitation of localized surface plasmon resonances (LSPRs) in metallic nanostructures. When incident light interacts with the free electrons in these nanostructures, it induces collective oscillations known as surface plasmons. These plasmons generate intense local electromagnetic fields, particularly at sharp edges, corners, or in small gaps between nanoparticles, commonly referred to as “hot spots” [21]. Molecules in close proximity to these hot spots experience greatly enhanced electromagnetic fields, leading to a significant increase in their Raman scattering cross-section (Figure 1A). The magnitude of this enhancement can be approximated by the fourth power of the local field intensity, resulting in enhancement factors that can reach 10^6^ to 10^8^, or even higher under optimal conditions. This extraordinary amplification enables the detection of molecules at extremely low concentrations, sometimes down to the single-molecule level.

The chemical enhancement mechanism, while generally considered less significant than its electromagnetic counterpart, can still contribute a factor of 10–100 to the overall SERS enhancement. This mechanism involves the formation of charge-transfer complexes between the adsorbed molecule and the metal surface [16,22]. The resulting changes in the electronic structure of the molecule can lead to an increase in its polarizability and, consequently, its Raman scattering cross-section (Figure 1B). There are probably chemical behaviors that increase the adsorption energy of molecules onto the metal surface, allowing the molecules to come closer to the metal surface and thereby enhancing the signal. Additionally, the metal surface can mediate photon-induced charge transfer processes, further contributing to the enhancement of Raman signals.

### 2.2. Noble Metal Nanoparticles in SERS

**Figure 2:**
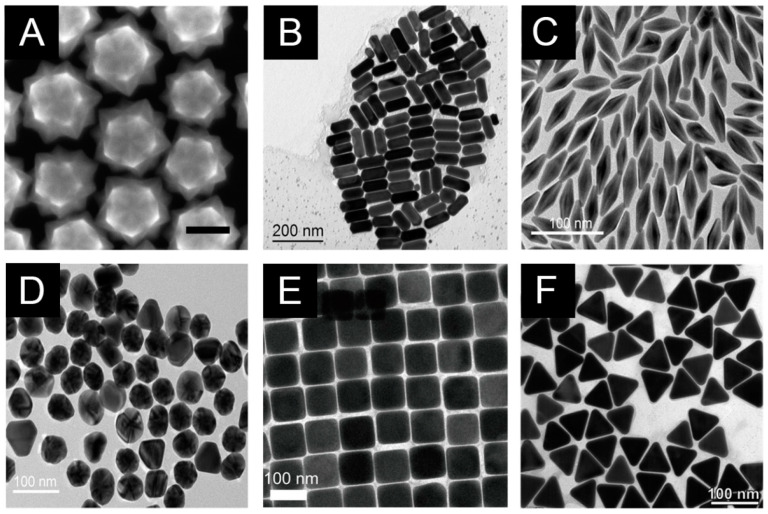
TEM images of monodispersed gold nanostars (**A**), gold nanorods (**B**), gold nano-bipyramids (**C**), spherical gold nanoparticles (**D**), gold nanocubes (**E**), and gold nanotriangles (**F**). Reprinted with permission from refs. [28,29,30,31,32]. Copyright Elsevier and ACS.

Noble metals, particularly gold and silver, have been the cornerstone of SERS substrates due to their exceptional plasmonic properties in the visible and near-infrared regions of the electromagnetic spectrum [23]. These metals exhibit strong plasmon resonances and relatively low losses, making them ideal candidates for SERS applications. Gold nanoparticles are widely used in SERS due to their excellent stability, biocompatibility, and tunable plasmon resonances [18,24,25]. By adjusting the size, shape, and aspect ratio of gold nanostructures, researchers can tailor their optical properties to match specific excitation wavelengths or resonate with particular molecular vibrations of interest [26,27]. The common gold nanostructures employed in SERS include spheres, rods, stars, and various anisotropic shapes (Figure 2) that offer high curvature and abundant hot spots.

Silver nanoparticles, while more prone to oxidation than gold, often provide even stronger SERS enhancements due to their superior plasmonic properties in the visible range. Silver nanostructures can generate more intense and confined electromagnetic fields, resulting in higher enhancement factors [33]. However, their chemical instability and potential toxicity can limit their applicability in certain biological and environmental sensing scenarios.

**Figure 3:**
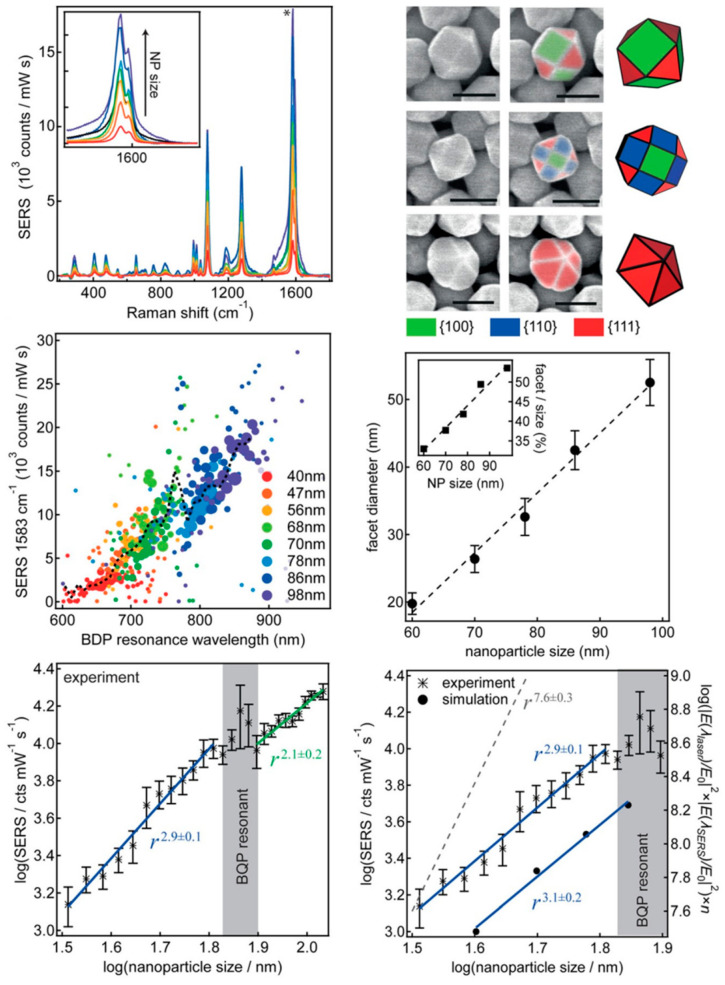
Correlation of SERS intensity and scattering resonance. Gold nanoparticle faceting and impact on SERS enhancement. Reprinted with permission from ref. [35]. Copyright 2016 ACS.

The size of noble metal nanoparticles plays a crucial role in determining their SERS performance. Nanoparticles (typically 10–50 nm) often exhibit stronger plasmonic fields and higher hot spot densities than nanoparticles (below about 10 nm), leading to greater SERS enhancements [34]. As particle size decreases below about 10 nm, increased electron scattering can dampen the plasmon resonance, potentially reducing SERS efficiency. Benz et al. [35] found that the relationship between particle size and SERS performance is complex and not simply linear. The study found that larger nanoparticles generally produce stronger SERS signals, but the increase is not as dramatic as might be expected based on particle size alone. Specifically, doubling the nanoparticle size from 50 to 100 nm increased the Raman signal intensity by a factor of about 5 on average. The SERS intensity scaled approximately with r^2.9^ (where r is the nanoparticle radius), which is lower than the r^7.6^ scaling observed for far-field scattering intensity (Figure 3). This discrepancy is attributed to the changing shape of larger nanoparticles, which become increasingly non-spherical and faceted as they grow in size. The facets spread out the confined optical field, reducing the maximum field enhancement possible compared to ideal spheres [36]. The researchers found two distinct scaling regions separated by a peak where the bonding quadrupolar plasmon mode is resonant with the Raman laser. Overall, using the largest possible nanoparticles produced the strongest SERS signals, but the enhancement was less than predicted by simple scaling laws due to the effects of particle shape. The study highlights that both size and shape play crucial roles in determining SERS performance of individual nanoparticles. The excitation wavelength of nanoparticles is also very important. It is necessary to consider the direction of their plasmon resonance based on their spectra, ensuring that the corresponding wavelength range is selected to achieve optimal signal enhancement [37,38]. In another work, Xiu et al. [34] revealed a positive relationship between silver nanoparticle size and SERS performance within the 35–65 nm range. As the particle size increased, the SERS signal strength improved, despite a decrease in surface number density. This enhancement is attributed to stronger LSPR coupling with the 633 nm excitation wavelength used. The increase in absorption and scattering cross-section of larger particles outweighed the reduction in surface coverage, resulting in overall stronger SERS signals. Finite element modeling supported these findings, showing a monotonic increase in SERS enhancement factor with particle size. Particularly for particle dimers, which create “hot spots” responsible for most of the SERS signal, the enhancement factor increased dramatically with particle size and decreased gap width.

**Figure 4:**
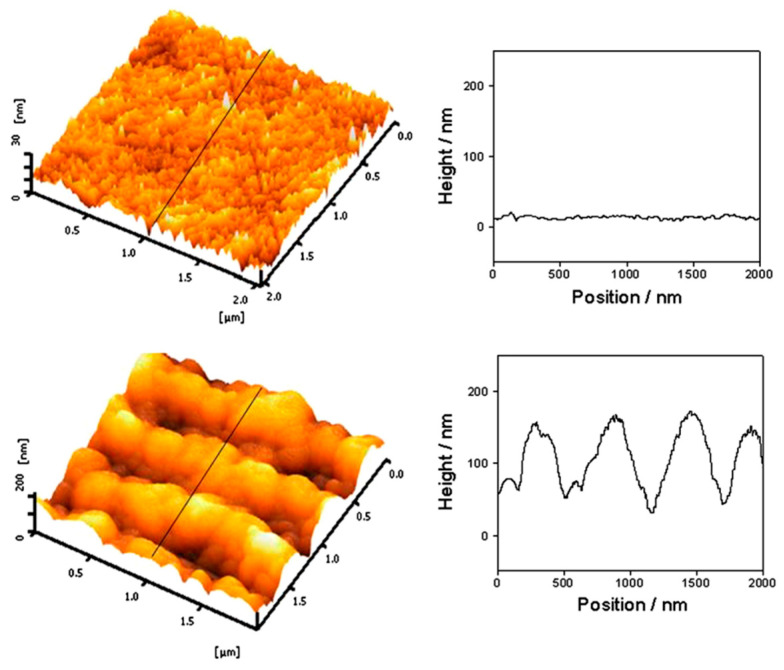
AFM images of the surface of Ag substrate before and after irradiating by femtosecond laser. Reprinted with permission from ref. [44]. Copyright 2011 Elsevier.

The shape of nanoparticles is another critical factor influencing SERS performance. Anisotropic nanostructures with sharp features, such as nanostars [39], nanotriangles [40], or nanoflowers [41], can concentrate electromagnetic fields at their tips or vertices, creating intense hot spots. Similarly, assemblies of nanoparticles that form small gaps or junctions between particles can generate exceptionally strong electromagnetic fields in these interparticle regions, leading to dramatic SERS enhancements [42]. Surface roughness and nano-texturing of noble metal surfaces also play important roles in SERS. Rough surfaces with nanoscale features can support localized plasmons and provide numerous adsorption sites for target molecules, enhancing both electromagnetic and chemical enhancement mechanisms [43]. Chang et al. [44] demonstrated a clear relationship between surface roughness and SERS performance. The nanostructured Ag surface fabricated by femtosecond laser exhibited significantly enhanced SERS activity compared to the untreated Ag substrate. AFM measurements revealed that the root mean square surface roughness increased from 2.9 nm for the untreated Ag surface to 43.8 nm for the laser-treated surface (Figure 4). This increased roughness corresponded to a 15-fold enhancement in SERS intensity for rhodamine 6G (R6G) detection. The laser treatment created periodic surface structures covered with random nanostructures, including nanocavities and nanospheres. These nanostructures likely provided “hot spots” for localized electromagnetic field enhancement, a key mechanism for SERS. The rougher surface also increased the effective surface area for molecular adsorption. Consequently, the nanostructured Ag surface demonstrated high SERS sensitivity, allowing for the detection of R6G at concentrations as low as 10^−8^ M, with a linear response range of 10^−8^ to 10^−4^ M. This strong correlation between increased surface roughness and enhanced SERS performance underscores the importance of surface nanostructuring in developing effective SERS substrates.

### 2.3. Advantages of Hybrid Nanomaterials for SERS

**Figure 5:**
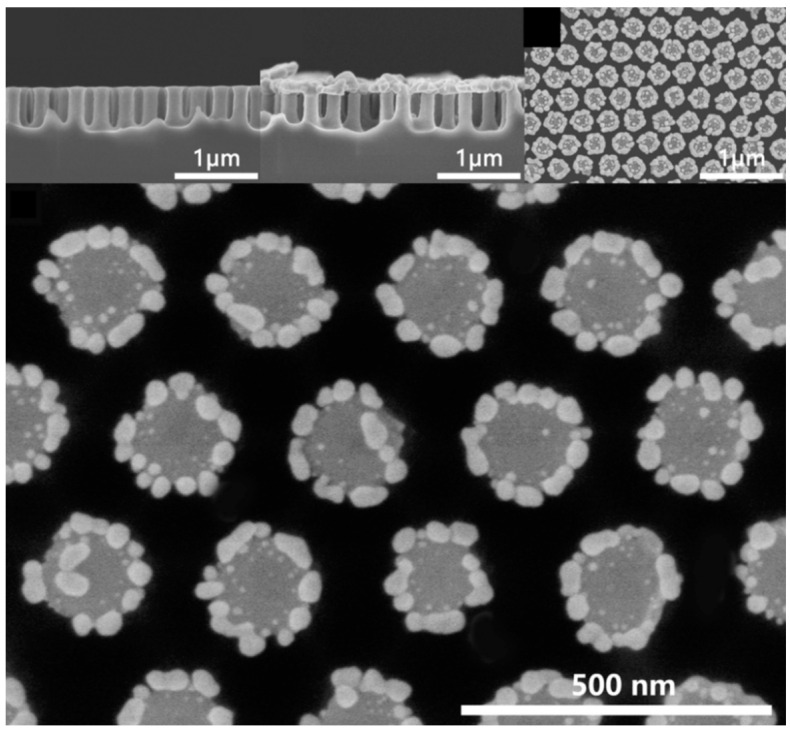
SEM image of the created Si nanopillars and the AgNPs formed on the Si nanopillars. Reprinted with permission from ref. [51]. Copyright 2017 ACS.

While noble metal nanoparticles have been the mainstay of SERS substrates, the field has recently witnessed a surge of interest in hybrid nanomaterials that combine noble metals with other functional materials [45,46]. These hybrid systems offer several advantages over traditional noble metal nanoparticles, addressing some of the limitations of pure metallic substrates and opening up new possibilities for SERS-based sensing. One of the primary advantages of hybrid nanomaterials is enhanced stability. Pure noble metal nanoparticles, especially silver, are prone to oxidation and aggregation, which can lead to reduced SERS activity over time. By incorporating noble metals into hybrid structures or coating them with protective layers, researchers can significantly improve the long-term stability and shelf-life of SERS substrates [47]. For instance, core-shell structures where a thin layer of gold encapsulates a silver core can combine the superior plasmonic properties of silver with the chemical stability of gold [48,49,50]. Hybrid nanomaterials also offer opportunities for improved reproducibility in SERS measurements. The controlled assembly of noble metal nanoparticles on supporting materials or within porous structures can lead to more uniform distributions of hot spots, reducing signal variability across the substrate. For example, Xu et al. [51] utilized a droplet-confined electroless deposition (DCED) method to create uniformly distributed AgNPs on hydrophobic silicon nanopillar arrays (Figure 5). By confining the deposition to the tops of the nanopillars, the method ensures that hot spots are formed in accessible locations with consistent spacing. This results in more uniform electromagnetic field distributions and improved reproducibility in SERS measurements. The hydrophobic nature of the substrate further enhances the concentration of analyte molecules at these hot spots. Consequently, the relative standard deviation of SERS measurements is reduced to as low as 3.40%, and a strong linear correlation (R^2^ = 0.998) is observed between analyte concentration and Raman intensity. This controlled assembly approach not only improves the uniformity of SERS substrates but also enhances their sensitivity, allowing for detection limits as low as 10^−11^ M for R6G.

**Figure 6:**
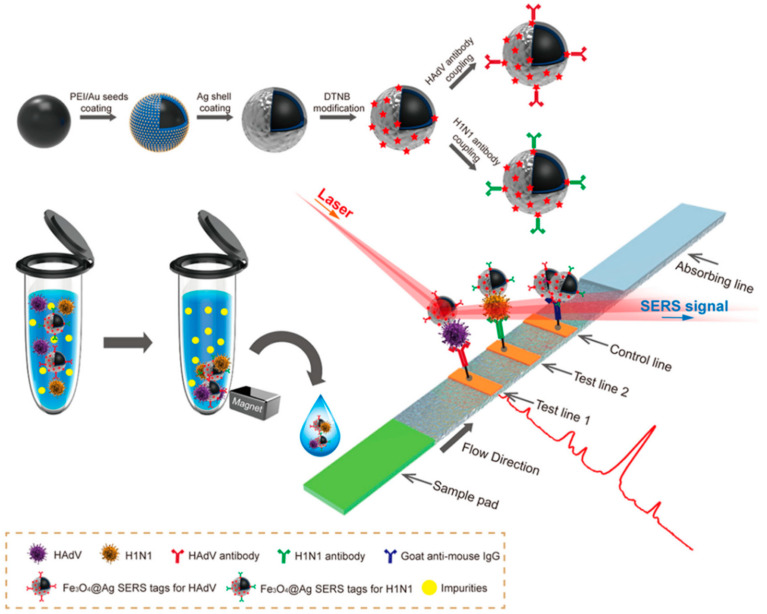
Synthetic route for antibody-modified Fe_3_O_4_@Ag magnetic tags and schematic diagram of the magnetic SERS Strip for detecting two respiratory viruses. Reprinted with permission from ref. [52]. Copyright 2019 ACS.

**Figure 7:**
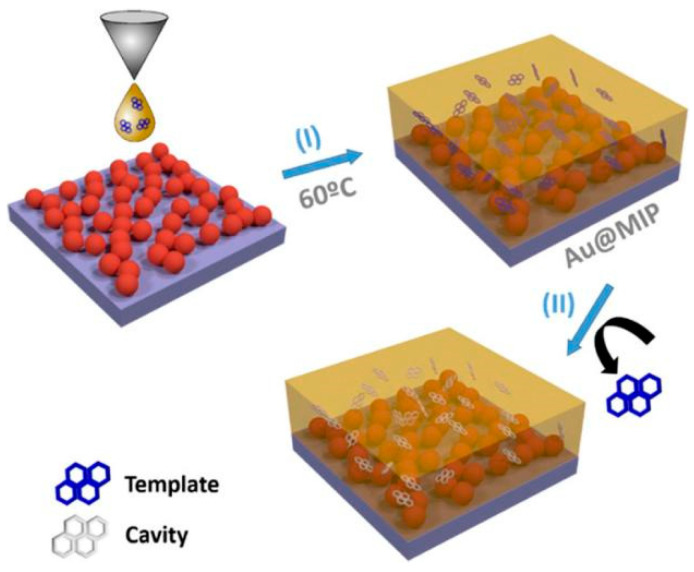
Schematic representation of Au@MIP fabrication process. Reprinted with permission from ref. [53]. Copyright 2020 ACS.

Another significant advantage of hybrid systems is the potential for multifunctionality. By combining noble metals with materials that possess complementary properties, researchers can create SERS substrates with additional functionalities beyond signal enhancement. For example, incorporating magnetic materials into the hybrid structure can enable the magnetic separation and concentration of analytes. Wang et al. [52] developed a SERS lateral flow immunoassay strip for the simultaneous detection of influenza A H1N1 virus and human adenovirus (HAdV) using Fe_3_O_4_@Ag nanoparticles as magnetic SERS tags. The magnetic tags served multiple functions: specifically recognizing and magnetically enriching target viruses in solution, and enabling SERS detection on the strip (Figure 6). This approach allowed for direct use on biological samples without pretreatment. The magnetic SERS strip achieved limits of detection of 50 pfu/mL for H1N1 and 10 pfu/mL for HAdV, 2000 times more sensitive than standard colloidal gold strips. It demonstrated good selectivity, stability over pH 5–9, and applicability in complex biological samples like blood and sputum. The magnetic enrichment capability of the Fe_3_O_4_@Ag tags enabled rapid separation of targets from complex matrices within 30 s. The use of magnetic SERS tags in lateral flow assays was a novel approach that significantly enhanced sensitivity and enabled the direct testing of complex clinical samples. The integration of molecularly imprinted polymers (MIPs) can enhance selectivity toward specific target molecules. Castro-Grijalba et al. [53] developed a SERS sensor for detecting polycyclic aromatic hydrocarbons (PAHs) by combining AuNP assemblies with MIP thin films (Figure 7). The researchers optimized the fabrication process to create uniform MIP coatings on gold substrates, using pyrene and fluoranthene as template molecules. The resulting Au@MIP sensors demonstrated remarkable sensitivity, achieving detection limits down to 1 nM for both pyrene and fluoranthene, which was 100 times lower than non-imprinted polymer sensors. The MIP coating enabled the selective trapping of target PAHs near the gold surface, enhancing SERS detection. Binding constants of 3.2 × 10^−6^ M for pyrene and 1.3 × 10^−6^ M for fluoranthene were determined. The sensors exhibited high selectivity, with pyrene-based Au@MIPs detecting only pyrene in mixed PAH solutions. The practical applicability was demonstrated by successfully detecting pyrene in spiked creek water and seawater samples, maintaining sensitivity down to 1 nM. This hybrid plasmonic-MIP approach overcame the limitations of traditional SERS and MIP sensors, offering a promising platform for ultrasensitive and selective detection of environmental pollutants.

**Figure 8:**
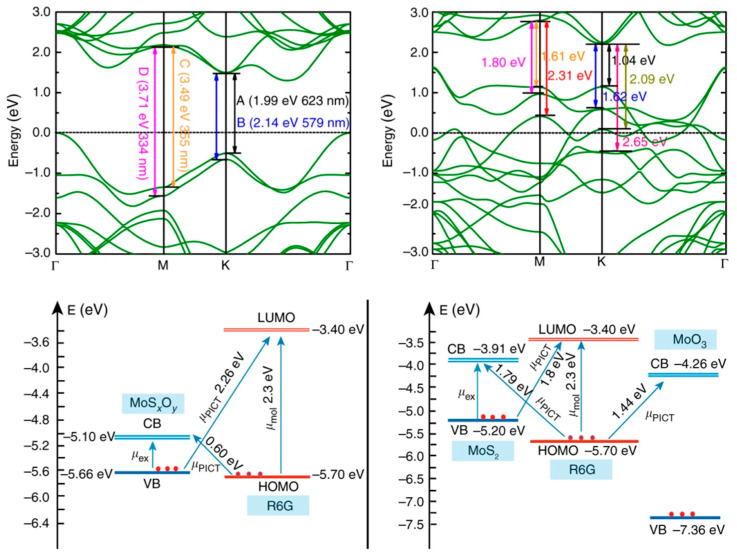
Energy-level diagrams illustrating the electronic transitions. The calculated band structures of MoS_2_ a and MoS_x_O_y_ taking Fermi level as the reference. Schematic energy-level diagrams of R6G on c MoS_x_O_y_ and d MoS_2_ and MoO_3_ with respect to the vacuum level. Reprinted with permission from ref. [56]. Copyright 2017 Nature.

Furthermore, hybrid systems offer opportunities to exploit synergistic effects between the noble metal and the supporting material [54,55]. For instance, semiconductor-metal hybrids can facilitate charge transfer processes that contribute to chemical enhancement mechanisms. Zheng et al. [56] found that oxygen incorporation in non-metal-oxide semiconductors like MoS_2_ can significantly enhance SERS signals by up to 100,000 times. This enhancement stems from the coupling of multiple resonances: charge transfer, molecular, and exciton resonances. Oxygen incorporation creates additional energy levels that facilitate charge transfer between the semiconductor and analyte molecules in resonance with incident photons. It also improves exciton resonances, allowing for stronger intensity borrowing to the charge-transfer resonance. By manipulating oxygen atoms in the semiconductor lattice through incorporation or extraction, the energy levels can be tuned to optimize both charge-transfer and exciton transitions for specific molecules (Figure 8). This approach provides new insights into the chemical mechanism of SERS enhancement in semiconductor-based substrates and demonstrates a way to engineer highly sensitive SERS materials without using noble metals. Similarly, graphene–metal hybrids can leverage the exceptional electronic properties of graphene to modulate plasmonic responses and enhance SERS signals. Using ultrafast optical spectroscopy, Wang et al. [57] demonstrated that electrons rapidly transfer from graphene to AgNPs in a graphene/Ag-NP hybrid structure. This electron transfer from graphene, acting as an electron reservoir, enhances plasmon excitations in the Ag-NPs, generating a stronger local electromagnetic field. The enhanced field contributes significantly to ultrasensitive SERS detection, achieving sensitivity as high as 10^−13^ M for R6G molecules. Finite-difference time-domain simulations confirmed the graphene-induced electric field enhancement. The vertical graphene/Ag-NP hybrid structure on silicon pyramids demonstrated excellent SERS performance, uniformity, and repeatability. The study clarifies graphene’s role in plasmon enhancement and electromagnetically enhanced SERS through the carrier transfer mechanism. This understanding opens up new opportunities for designing ultrasensitive SERS substrates and other plasmonic devices. The graphene–metal hybrid approach leverages graphene’s high carrier mobility and broadband linear dispersion to create more effective plasmonic nanostructures, potentially leading to improved chemical and biological sensors, as well as other applications requiring strong light-matter interactions.

The integration of noble metals with porous materials, such as metal–organic frameworks or mesoporous silica, can create three-dimensional SERS-active structures with high surface areas and abundant hot spots. For example, Zhang et al. [58] developed a method for detecting complex samples by combining SERS with thin-layer chromatography (TLC). The researchers fabricated an enhanced TLC (eTLC) plate using metal-organic framework MIL-101-modified AuNPs as a separable SERS substrate. This innovative approach allowed for the detection of all separated components, including overlapping and invisible compounds, through a point-by-point SERS scan along the developing direction. The eTLC-SERS method demonstrated excellent sensitivity, reproducibility, and stability, with relative standard deviations below 16% for 100 repeated tests within one batch and under 18% for 20 different batches. The substrate maintained its performance after more than three months of storage. Compared to conventional TLC-SERS, the eTLC-SERS method provided six-times-stronger SERS signals (52,724 vs. 8537) and achieved optimal detection within 5 s. The technique successfully detected 1% (*w*/*w*) adulterants in botanical dietary supplements and distinguished overlapping compounds. By integrating noble metals with the porous MIL-101 framework, they created a 3D structure that accommodated more SERS-active sites and improved adsorption, resulting in enhanced sensitivity and stability for complex sample analysis. Lu et al. [59] introduced an approach for creating metallic-surface nanopatterns using MOFs as templates. The researchers discovered that MOF templates could guide the electrochemical growth of metals exclusively underneath them, resulting in precise nanopatterns. By adjusting electrolyte concentrations, they could switch between guiding and molding growth modes. The guiding mode was enabled by rapid ion transport through MOF nanochannels, confirmed by both experiments and molecular dynamics simulations. Using this method, they fabricated silver nanotriangle arrays that exhibited strong SERS performance, detecting R6G molecules at concentrations as low as 10 nM. The MOF templates were reusable, allowing for the cost-effective and repeatable production of identical metallic nanopatterns. This technique was demonstrated with different MOFs and metals, showcasing its versatility. The integration of noble metals with porous MOF materials resulted in highly ordered nanostructures with enhanced plasmonic properties. These structures can significantly increase the number of adsorption sites for target molecules and provide a more favorable environment for molecular interactions and signal enhancement.

In the context of urinary metabolite analysis, hybrid nanomaterials offer several specific advantages. The complex composition of urine, with its high salt content and diverse range of metabolites, can pose challenges for traditional SERS substrates. Hybrid materials can be designed to mitigate interfering effects from the urine matrix, improve selectivity toward specific metabolites of interest, and enhance the overall robustness of the SERS-based sensing platform.

## 3. Fabrication and Characterization of Noble Metal Nanohybrids for SERS

The development of noble metal nanohybrids for SERS applications has seen remarkable progress in recent years, with various innovative fabrication techniques and characterization methods emerging [60]. These hybrid materials combine the plasmonic properties of noble metals with the unique characteristics of other materials, resulting in SERS substrates with enhanced performance and functionality. This section explores the fabrication and characterization of several key categories of noble metal nanohybrids, highlighting their potential for SERS-based urinary metabolite analysis.

### 3.1. Carbon-Based Hybrids (Graphene and Carbon Nanotubes)

**Figure 9:**
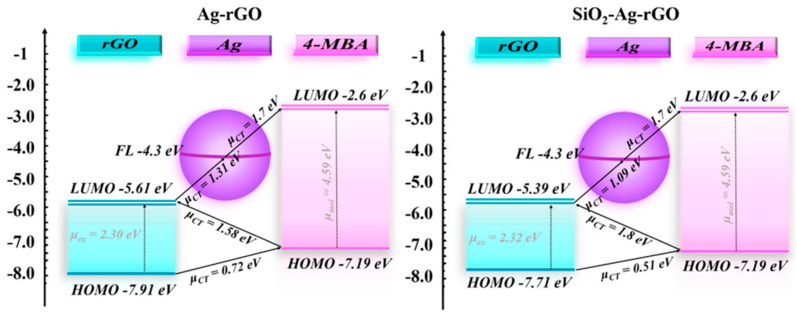
The proposed mechanisms of the CT process in the Ag–rGO system and SiO_2_–Ag–rGO system combined with 4-MBA molecules. Reprinted with permission from ref. [62]. Copyright 2021 ACS.

Carbon-based materials, particularly graphene and carbon nanotubes (CNTs), have gained significant attention as components in noble metal nanohybrids for SERS. These materials offer exceptional electrical and thermal conductivity, high surface area, and unique optical properties that can synergistically enhance SERS performance when combined with noble metals. Graphene–noble metal hybrids are typically fabricated through various methods, including in situ reduction, self-assembly, and physical deposition [61]. One common approach involves the reduction of graphene oxide in the presence of noble metal precursors, resulting in the simultaneous formation of metal nanoparticles on graphene sheets. Guo et al. [62] investigated a SiO_2_-Ag-rGO composite structure for enhanced SERS activity. The researchers synthesized the composite using a simple wet chemical method, decorating Ag nanoparticles and SiO_2_ onto rGO surfaces. They found that introducing Si atoms controlled both the plasmon effect of Ag nanoparticles and the defect concentration in rGO. The increased defects created a metastable state in rGO, facilitating charge separation and transfer. By adjusting the defect concentration, they achieved charge transfer resonance coupling (Figure 9). The composite exhibited ultrahigh SERS activity, with the charge transfer contribution (ρCT) reaching 0.31 under 633 nm laser excitation. The researchers also established a relationship between defect concentration and ρCT, demonstrating how structural disorder affects the optical properties of rGO-based composites. This approach of reducing graphene oxide in the presence of noble metal precursors resulted in the formation of Ag nanoparticles on graphene sheets with an average diameter of 23.4 ± 4.4 nm. This method allows for the precise control over nanoparticle size and distribution. Alternatively, pre-synthesized noble metal nanoparticles can be attached to graphene surfaces through electrostatic interactions or covalent bonding [63].

**Figure 10:**
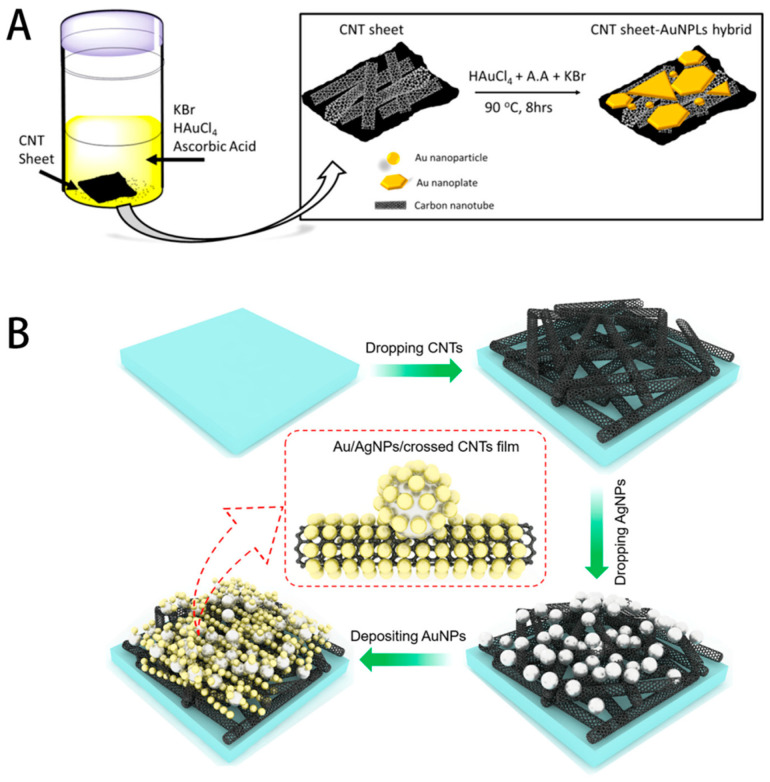
Schematic illustration of the one-pot synthesis of AuNPLs on CNT sheet (**A**) and the synthetic procedure of Au/AgNP/crossed CNT substrate (**B**). Reprinted with permission from refs. [66,67]. Copyright 2017 ACS and 2021 MDPI.

Carbon nanotube-noble metal hybrids are often prepared by decorating CNT surfaces with metal nanoparticles [64,65]. This can be achieved through electroless deposition, where metal ions are reduced directly on the CNT surface, or by attaching pre-formed nanoparticles to functionalized CNTs. For example, Xin et al. [66] demonstrated an one-pot synthesis method for creating gold nanoplates (AuNPLs) directly on CNT, forming a composite that served as an effective flexible substrate for SERS (Figure 10A). The process involved a hydrothermal reduction technique where bromine ions facilitated the anisotropic growth of the AuNPLs. This composite structure was designed to enhance SERS performance by leveraging the unique properties of the AuNPLs, such as their large surface area, sharp edges, and nanoscale gaps between neighboring plates. The composite substrate exhibited exceptional sensitivity capable of detecting the analyte R6G at concentrations as low as 10^−7^ M. Wei et al. [67] focused on the synthesis of a three-dimensional (3D) Au/Ag nanoparticle(NP)/crossed carbon nanotube film SERS substrate (Figure 10B). The experimental results show that the proposed SERS substrate possesses an excellent sensitivity of 10^−12^ M R6G, an enhancement factor of 1.60 × 10^9^, and a good signal reproducibility (the relative standard deviation is ~6.03%). The unique tubular structure of CNTs provides a high surface area for nanoparticle attachment and can create interesting three-dimensional SERS-active architectures.

The characterization of these carbon-based hybrids typically involves a combination of microscopy and spectroscopy techniques [68,69]. Transmission electron microscopy (TEM) and scanning electron microscopy (SEM) are essential for visualizing the morphology and distribution of metal nanoparticles on the carbon surfaces [70]. X-ray diffraction (XRD) and X-ray photoelectron spectroscopy (XPS) provide information about the crystalline structure and chemical composition of the hybrids [71]. Raman spectroscopy itself is a powerful tool for characterizing these materials, as it can reveal the quality and degree of functionalization of the carbon structures.

### 3.2. Metal–Organic Framework (MOF) Hybrids

**Figure 11:**
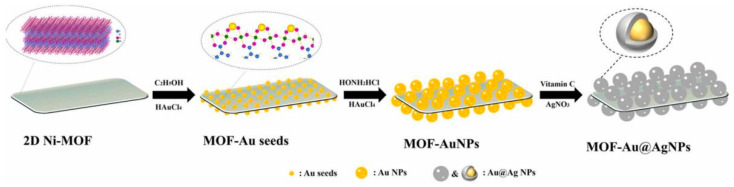
Illustration of the preparation of 2D Ni-MOF-Au@AgNPs composite. Reprinted with permission from ref. [73]. Copyright 2022 Elsevier.

Metal–organic frameworks (MOFs) have emerged as promising platforms for creating noble metal nanohybrids for SERS. MOFs are crystalline materials consisting of metal ions or clusters coordinated to organic ligands, forming porous three-dimensional structures [72]. The high surface area, tunable pore size, and diverse chemistry of MOFs make them excellent candidates for incorporating noble metal nanoparticles and creating SERS-active substrates. The fabrication of MOF–noble metal hybrids can be achieved through several strategies. One approach involves the in-situ growth of noble metal nanoparticles within the pores or on the surface of pre-formed MOFs. This can be accomplished by impregnating the MOF with metal precursors followed by reduction. Lai et al. [73] developed a SERS substrate by in situ growth of core-shell Au@Ag nanoparticles onto two-dimensional Ni-MOF nanosheets (Figure 11). This approach allowed for a dense and uniform distribution of nanoparticles, enhancing the electromagnetic field and improving SERS activity. The Ni-MOF-Au@AgNPs substrate demonstrated high enhancement factors of 2.2 × 10^6^ for thiram, 3.7 × 10^5^ for diquat, and 9.5 × 10^5^ for paraquat, with detection limits of 87.1 μg/L, 188.8 μg/L, and 8.9 μg/L, respectively. Another method is the simultaneous synthesis of MOFs and metal nanoparticles, where the metal ions serve both as nodes for MOF formation and precursors for nanoparticle growth. Zhang et al. [74] focused on the synthesis of a composite material made from AuNPs and a MOF-74 to detect 4-nitrothiophenol using SERS. They successfully synthesized core-shell nanoparticles with a gold core and MOF-74 shell through the one-pot synthesis method. The detection method showed a linear response in the concentration range of 0.10–10 μM and a low detection limit of 69 nM. The integration of MOFs and metal nanoparticles provided enhanced stability and reusability, making the composite a promising candidate for real-time SERS monitoring of reaction processes. It is worth noting that while noble metal nanohybrids MOFs materials are a novel material system, thorough biocompatibility and toxicity assessments must be conducted for their use in portable and clinical detection to ensure their safety for human use. Therefore, specific toxicity issues need to be analyzed in detail based on the particular MOF materials and their application environments [75].

Some innovative techniques involve using MOFs as sacrificial templates. In this approach, MOFs are first synthesized with desired morphologies, then noble metals are deposited on their surfaces. Subsequently, the MOF structure is selectively removed, leaving behind a porous noble metal structure that retains the original MOF morphology. For example, above mentioned work conducted by Lu et al. [59] explored the use of MOFs as templates in electrochemical lithography to create metallic surface nanopatterns for SERS sensing applications. They developed a method using MOF microparticles to guide the electrochemical growth of metallic films exclusively beneath the templates, revealing a guiding growth mode. This approach enabled the precise formation of metallic nanopatterns, significantly enhancing their structural diversity and application potential. The MOF templates were reusable, allowing for the cost-effective fabrication of identical nanopatterns across different substrates.

### 3.3. Silicon-Based Hybrids

**Figure 12:**
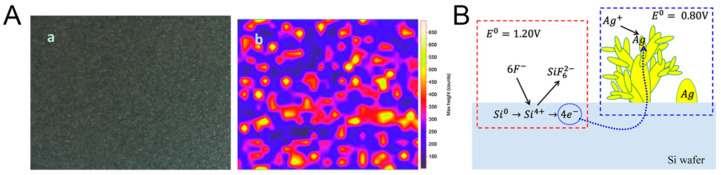
Optical image of the Ag/pSi SERS substrate (**a**) and SERS mapping (**b**) for 10^−5^ M R6G on the area (**A**). Schematics of the Galvanic replacement reaction of silver dendritic nanoforests on silicon (Ag-DNFs/Si) (**B**). Reprinted with permission from refs. [76,78]. Copyright 2019 Elsevier and 2020 MDPI.

Silicon-based materials, including porous silicon and silicon nanowires, have been extensively explored as substrates for noble metal nanohybrids in SERS applications [76]. The combination of silicon’s semiconductor properties with the plasmonic effects of noble metals can lead to interesting optical phenomena and enhanced SERS performance [77]. Fabrication of silicon-based noble metal hybrids often begins with the creation of nanostructured silicon surfaces. This can be achieved through electrochemical etching to produce porous silicon or vapor-liquid-solid growth for silicon nanowires. Noble metals are then deposited onto these structures using techniques such as sputtering, thermal evaporation, or electroless deposition. For example, Yue et al. [76] synthesized a stable silicon-based SERS substrate by sputtering silver onto porous silicon (pSi) created through electrochemical etching. Characterized by SEM, the substrate demonstrated uniform shape and size, with stable SERS activity (Figure 12A). The study evaluated its performance using R6G as a probe and confirmed its signal uniformity through mapping measurements. The Ag/pSi substrate successfully detected R6G at concentrations as low as 10^−8^ M, showcasing high sensitivity. The process of this work is simple and highly reproducible, making it suitable as a method for mass-producing substrates. However, industrialization requires a large amount of silicon-based materials, which also involves many cost control issues.

An interesting approach involves the galvanic displacement reaction, where silicon acts as a reducing agent for noble metal ions, resulting in the spontaneous deposition of metal nanoparticles on the silicon surface. This method can produce unique hierarchical structures with high SERS activity. Huang et al. [78] synthesized silver dendritic nanoforests (Ag-DNFs) on silicon substrates using a fluoride-assisted galvanic replacement reaction (FAGRR) (Figure 12B). The FAGRR method allowed for the growth of Ag-DNFs with a large surface area and high aspect ratio.

### 3.4. Semiconductor Hybrids

Semiconductor–noble metal hybrids represent an intriguing class of materials for SERS, as they can combine plasmonic enhancement with photocatalytic and charge-transfer effects [79]. The common semiconductor materials used in these hybrids include titanium dioxide, zinc oxide, and various metal chalcogenides.

**Figure 13:**
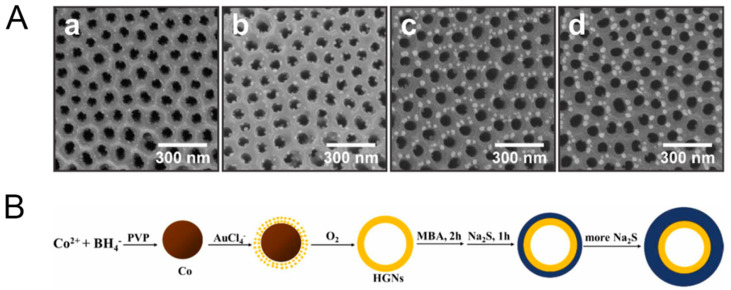
Top-view FESEM images for TiO2-NTFs exposed for different times to UVC radiation: 5 (**a**), 15 (**b**), 30 (**c**) and 60 min (**d**); with pre-immersion treatment in 50 mM AgNO_3_ (**A**). Schematic diagram for the synthesis of HGNs and HGNs-MBA@ZnS with well-controlled core morphology and tshell (**B**). Reprinted with permission from ref. [80,81]. Copyright 2024 Elsevier.

Fabrication of semiconductor-noble metal hybrids can be achieved through various methods. One approach involves the growth of semiconductor nanostructures followed by the deposition of noble metal nanoparticles. For instance, TiO_2_ nanotubes can be prepared by anodization, followed by the photoreduction of noble metal precursors on their surface. For example, Broens and Pérez [80] synthesized TiO_2_ nanotube films (TiO_2_-NTFs) through anodization, followed by photoreduction to decorate their surfaces with AgNPs. By varying the UVC exposure time and AgNO_3_ concentration, they achieved controlled nucleation and growth of Ag nanoparticles. The optimal conditions led to Ag nanoparticles with sizes between 20 and 30 nm and a surface density of approximately (0.9–2.4) × 10^10^ nanoparticles/cm^2^ (Figure 13A). These parameters were crucial in enhancing the SERS performance, as they facilitated the creation of numerous hot-spots necessary for significant Raman signal enhancement. The study also found that higher concentrations of AgNO_3_ could inhibit the water-assisted crystallization mechanism, preserving the nanotubular structure of TiO_2_ and maintaining its amorphous nature. Another strategy is the simultaneous formation of semiconductor and noble metal components through co-precipitation or hydrothermal synthesis. Yang et al. [81] synthesized AgNPs decorated on ZnAl layered double hydroxides (LDHs) using a one-pot solvothermal growth method. By co-precipitating metal and semiconductor components, they achieved a synergistic effect that improved both electromagnetic and charge transfer mechanisms. The Ag-ZnAl hybrid demonstrated a detection limit as low as 1 × 10^−10^ M for 4-nitrobenzenethiol (4-NBT), with an enhancement factor of 1.7 × 10^5^. This was attributed to the densely distributed AgNPs creating abundant “hot spots” and a higher concentration of oxygen vacancies promoting charge transfer.

Core-shell structures, where either the semiconductor or the noble metal forms the core, are particularly interesting for SERS applications. These can be fabricated through seed-mediated growth or layer-by-layer deposition techniques. For example, Wang et al. [82] synthesized hollow AuNPs (HGNs) coated with a ZnS shell to explore their potential in enhancing SERS. The team successfully prepared HGNs-MBA@ZnS with varying ZnS shell thicknesses from 1 to 19 nm using a straightforward two-step method (Figure 13B). They discovered that the SERS intensity was highly dependent on the shell thickness, with the 2 nm shell providing the most significant enhancement, increasing the electric field intensity by about 33 times compared to the incident field. This enhancement was attributed to the near-field effects and plasmon-induced interfacial charge transfer transitions.

### 3.5. Polymer-Based Hybrids

**Figure 14:**
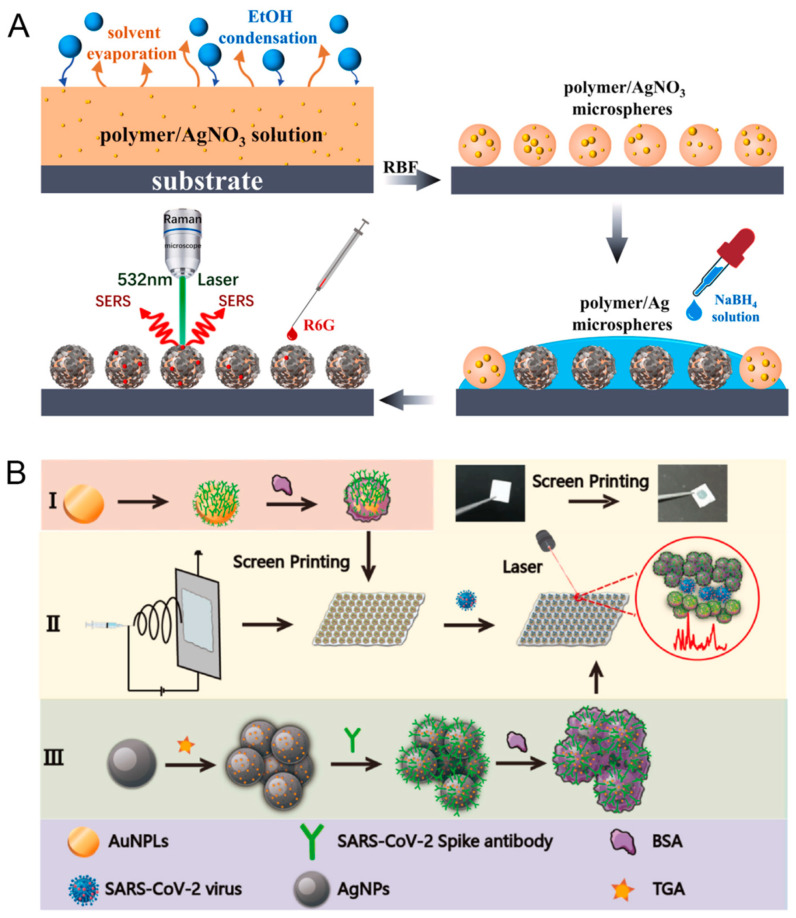
The schematic representation showing the fabrication of polymer/Ag core-shell microspheres and SERS application (**A**). Schematic diagram of the fabrication SERS-active substrate. (**B**): preparation of AuNPLs capture probe (**I**), preparation of PLA fiber flexible substrate (**II**), preparation of SERS nanotags (**III**). Reprinted with permission from refs. [85,86]. Copyright 2022 Elsevier and 2024 Elsevier.

Polymer–noble metal nanohybrids offer unique opportunities for SERS substrate design due to the versatility and functionalization capabilities of polymers. These hybrids can range from noble metal nanoparticles embedded in polymer matrices to more complex structures like polymer-coated noble metal nanoparticles or polymer nanofibers decorated with noble metals [83]. Fabrication methods for polymer-based hybrids are diverse and can be tailored to the specific polymer and desired structure. The in situ reduction of noble metal precursors within polymer solutions or matrices is a common approach, where the polymer acts as both a stabilizing agent and a template for nanoparticle formation. For example, Viriyakitpattana et al. [84] introduced a method for creating a plasmonic paper using a layer-by-layer assembly of biopolymers, chitosan, and alginate, to facilitate the in situ growth of AuNPs on filter paper. The double-layered biopolymer coating significantly enhanced the reduction properties, resulting in a high density of AuNPs, which improved the SERS performance. The substrate achieved an enhancement factor of 5.7 × 10^10^ and demonstrated a low limit of detection of 1.37 × 10^−12^ M for 4-mercaptobenzoic acid. Li et al. [85], researchers developed a method for fabricating polymer/Ag core-shell microspheres using the reverse breath figure (RBF) method combined with in situ reduction. Unlike traditional methods that require pre-prepared polymer microspheres, this technique allows for the one-step formation of core-shell structures by casting a solution of star-shaped POSS amphiphilic block copolymers and AgNO_3_ under ethanol vapor (Figure 14A). The AgNO_3_ is reduced in situ to form AgNPs on the polymer surface, resulting in microspheres with excellent SERS capabilities. Electrospinning is another powerful technique for creating polymer nanofibers, which can then be decorated with noble metal nanoparticles through post-processing steps. Sun et al. [86] developed a flexible polylactic acid (PLA) membrane using electrospinning to enhance the detection of respiratory viruses, such as SARS-CoV-2, through SERS. The electrospinning technique allowed for the creation of a flexible, high-surface-area substrate that significantly improved the adhesion and sensitivity of the detection probes compared to rigid substrates. This method involved screen printing gold nanoplates and antibodies onto the PLA membrane, forming a SERS-active substrate. AgNPs labeled with thioglycolic acid were used as SERS nanotags (Figure 14B). The study demonstrated that the sensor could detect the SARS-CoV-2 virus at concentrations as low as 10 transduction units per milliliter (TU/mL), offering a rapid, non-invasive diagnostic tool for early detection of respiratory infections. Compared to the high costs of silicon-based materials, electrospinning can be considered a low-cost manufacturing substrate technology. Its advantages include simple equipment and materials, high yield, low energy consumption, and flexibility, making it particularly advantageous for large-scale production.

## 4. SERS Detection of Urinary Metabolites Using Noble Metal Nanohybrids

The application of noble metal nanohybrids for SERS-based detection of urinary metabolites has shown remarkable progress in recent years. These advanced materials have enabled highly sensitive and specific detection of various metabolites, offering potential for non-invasive diagnostics and health monitoring. This section explores the SERS detection of key urinary metabolites using noble metal nanohybrids, highlighting recent advances and discussing the unique advantages these hybrid materials bring to metabolite analysis.

### 4.1. Uric Acid

**Figure 15:**
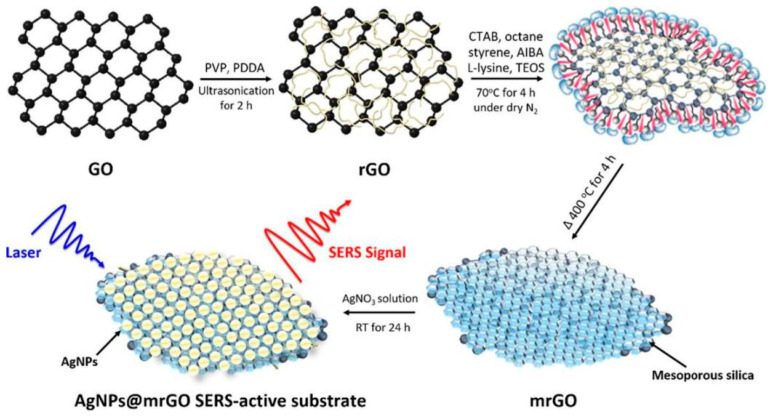
Schematic diagrams of AgNPs@mrGO SERS-active substrate. Reprinted with permission from ref. [90]. Copyright 2020 Elsevier.

Uric acid is a crucial biomarker for various physiological and pathological conditions, including gout, kidney disorders, and metabolic syndrome. The detection of uric acid using SERS has been significantly enhanced by the development of noble metal nanohybrids [87]. These hybrid materials offer improved sensitivity and selectivity compared to traditional SERS substrates [88]. One notable approach involves the use of graphene-silver nanoparticle hybrids for uric acid detection. The synergistic effect between graphene’s excellent adsorption properties and silver’s strong plasmonic enhancement results in a highly sensitive SERS platform. For example, Juang et al. [89] developed a floating SERS substrate using AgNPs embedded on poly(diallyldimethylammonium chloride) modified GO nanosheets. This novel substrate aimed to detect biomolecules and clinical uremic toxins, particularly in patients with chronic kidney disease (CKD), where the detection of uremic toxins like uric acid is crucial. The floating SERS substrate demonstrated high sensitivity and rapid detection capabilities. Specifically, for uric acid, the detection limit was about 2.5 × 10^−5^ M, with a linear detection range between 1 × 10^−4^ and 4 × 10^−4^ M. This sensitivity level is adequate for clinical applications, as it aligns with the typical concentration range found in healthy individuals. The substrate’s ability to provide label-free and ultrasensitive detection positions it as a promising tool for real-time clinical diagnostics and biotechnological applications, offering significant potential for improving the monitoring and management of CKD patients. In another work [90], they developed a SERS-active substrate using AgNPs embedded on mesoporous-silica modified rGO (mrGO) nanosheets (Figure 15). This substrate was designed for the sensitive detection of uremic toxins and parathyroid hormone (PTH). The AgNPs@mrGO substrate demonstrated a significant enhancement in signal-to-background ratio, showing a 6.9-fold increase compared to previous substrates. The study emphasized its application in detecting uric acid, achieving an LOD below 10^−6^ M, which is notably lower than the normal concentration range in healthy individuals.

Another innovative system employs gold nanoparticles decorated on semiconductors. Li et al. [91] reported a SERS-based non-enzymatic detection system for uric acid using Au/CeO_2_ nanorods. Au/CeO_2_ nanorods were synthesized through hydrothermal methods and characterized by their high catalytic activity and SERS performance. The system demonstrated a wide detection range with a remarkably low limit of detection at 3.29 × 10^−10^ M. It effectively detected uric acid in human urine samples, showcasing its potential for practical biomedical applications. Peng et al. [92] developed Au-Ag@MnO_2_ NPs for dual-mode sensing of uric acid, utilizing label-free SERS and plasmon-enhanced electroreduction (ER) techniques. These NPs exhibited inherent SERS activity without additional Raman molecule modification and two ER peaks at 0.5 V and 0.1 V, corresponding to Mn^4+^ and Ag^+^ reduction processes, respectively. Under LSPR excitation, the ER peak at 0.5 V was enhanced by 1.23 times, while the 0.1 V peak remained unchanged. The presence of H_2_O_2_ triggered MnO_2_ shell decomposition, exposing the Au-Ag NPs and resulting in “turn on” SERS signals and “turn off” ratiometric electrochemical responses. The dual-mode sensor achieved LODs for uric acid of 7.18 nM with SERS and 4.68 nM with electrochemical signals. Table 1 shows the recent reported SERS-based sensor for uric acid detection. It is important to note that Wang et al. [93] combined the photoinduced electron transfer properties of g-C_3_N_4_ with the plasmonic characteristics of gold, resulting in a material that achieved a detection limit for uric acid of 10^−11^ M, which is one of the few good works in the field of SERS. As uric acid is typically detected in urine, its concentration in the urine environment primarily ranges from 10^−5^ to 10^−3^ M. Therefore, we should focus more on the practical detection needs rather than solely pursuing the detection limit.

### 4.2. Urea

**Figure 16:**
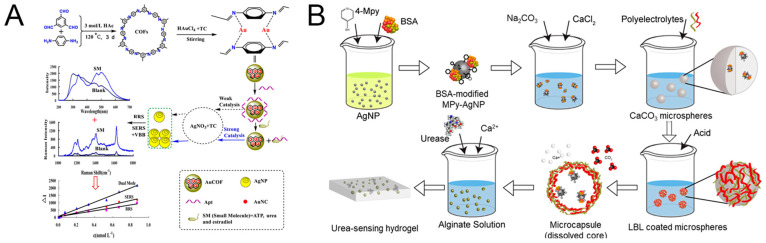
Principle of AuCOF preparation and aptamer-regulated AuCOF catalyzed RRS/SERS coupled dual-mode assay for small molecule (**A**). Synthesis of bovine serum albumin (BSA)-modified silver nanoparticles capped with 4-mercaptopyridine (AgNP-MPy), microcapsules containing BSA-modified MPy-AgNPs, and the urea sensing hydrogel (**B**). Reprinted with permission from refs. [100,103]. Copyright 2020 Elsevier and 2019 MDPI.

Urea, the most abundant organic compound in urine, serves as an important indicator of renal function and protein metabolism. The detection of urea using SERS has been challenging due to its weak Raman scattering cross-section [99]. However, noble metal nanohybrids have opened new avenues for sensitive urea detection. Yao et al. [100] developed a dual-mode SERS and Rayleigh scattering (RRS) aptasensor for detecting trace organic molecules, specifically targeting small molecules like urea. The aptasensor utilized gold nanocluster-doped covalent–organic frameworks (AuCOF) to enhance the catalytic performance in detecting molecules through SERS and RRS (Figure 16A). The AuCOF demonstrated strong catalytic activity, significantly improving the sensitivity and accuracy of the detection method. For urea detection, the system provided a linear range of 0.13 to 3.33 nM with a regression equation of ΔI = 879.9 C + 11 and a correlation coefficient of 0.9958, achieving a detection limit of 0.01 nM. Chamuah et al. [101] developed a SERS substrate using Blu-ray DVDs to detect albumin, creatinine, and urea in urine. The substrate efficiently guided LSPR fields of gold nanoparticles within the DVD’s nanochannels, enhancing Raman signal detection. This method achieved a minimum detectable concentration of 0.6 μg/mL for urea. Notably, the SERS substrate demonstrated high sensitivity, with LOD for urea being 0.084 μg/mL. Mukanova et al. [102] explored a cost-effective and straightforward method for detecting urea using a hybrid substrate of AuNPs on untreated aluminum foil. The method demonstrated superior detection limits compared to traditional substrates. For urea detection in artificial urine, the AuNPs on aluminum foil substrate achieved a LOD of 24 mM and a limit of quantification (LOQ) of 26 mM. These values were comparable to those obtained with gold film substrates but at a lower cost. The aluminum foil substrate exhibited a strong semi-logarithmic trendline with an R^2^ value of 0.98, indicating high reliability in quantifying urea concentrations within the physiological and pathophysiological range (0.03–0.15 M). Quinn et al. [103] reported a sensor utilizing hydrogel microdomains encapsulating functionalized AgNPs for SERS to measure urea levels. AgNPs, functionalized with 4-mercaptopyridine and stabilized with BSA, were used due to their high SERS amplification (Figure 16B). The sensor demonstrated a sensitive Raman scattering response over a pH range of 6.5 to 9.7. By incorporating urease into the hydrogel, the sensor could distinguish urea concentrations of 0, 0.1, 1, and 10 mM. BSA played a crucial role in preventing nanoparticle aggregation, enhancing the sensor’s sensitivity and stability. Table 2 shows the recent reported SERS-based sensor for urea detection. One particular study has provided us with new insights. Juang et al. [104] combined electrochemistry with SERS, generating an electric field around AuNPs through electrochemistry to create specific binding sites for urea, thereby enhancing detection capability. They developed a combined approach of the two detection methods, which complement each other to achieve better results.

### 4.3. Creatinine

**Figure 17:**
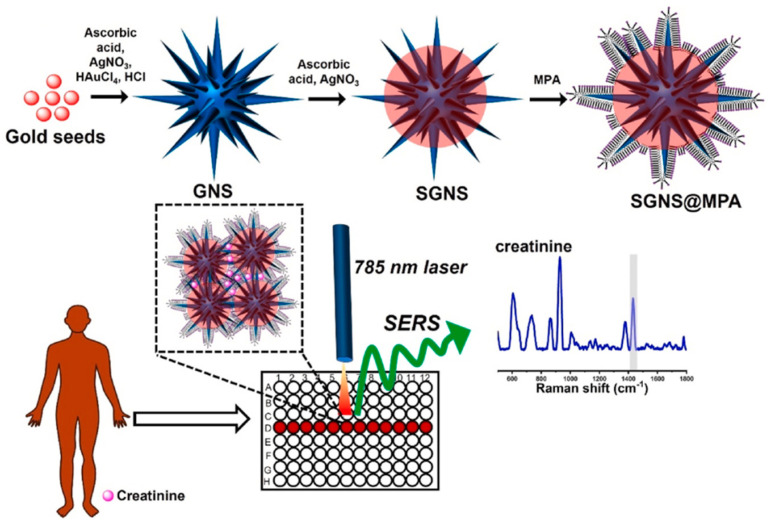
Schematic representation of the SGNS@MPA synthesis and microplate-based SERS detection platform for creatinine detection in human saliva. Reprinted with permission from ref. [113]. Copyright 2024 Elsevier.

Creatinine, a breakdown product of creatine phosphate in muscle metabolism, is widely used as a marker for renal function. The development of noble metal nanohybrids has significantly improved the SERS-based detection of creatinine in urine samples [110]. For example, Jiang et al. [111] developed an Au@MIL-101(Fe) composite as a novel substrate for the sensitive detection of creatinine in urine using SERS. The composite was synthesized through the in situ growth of gold nanoparticles within a MOF, MIL-101(Fe), which enhanced the local surface plasmon resonance effect, crucial for SERS. This method enabled the detection of creatinine with a LOD as low as 0.1 μM and showed a linear detection range from 1 μM to 100 μM. The research highlighted the composite’s ability to selectively enrich and detect creatinine, even in complex urine samples, with recovery rates ranging from 82.3% to 99.1%. Zhang et al. [112] developed hexagonal boron nitride/gold nanocomposites (h-BN/Au NCs) for the ultra-sensitive detection of creatinine molecules using SERS spectroscopy. The goal was to create a highly sensitive SERS substrate by fabricating h-BN/Au NCs with controllable gold compositions ranging from 0 to 1.85%. The optimal composition was found to be 1.68% gold, which provided the highest SERS activity. The study established well-defined linear relationships between SERS signal intensities and creatinine concentrations (10^−2^–10^−6^ M), offering precise assessments for pathological diagnostics. Atta and Vo-Dinh [113] developed a sensitive solution-based SERS platform to detect creatinine. This approach utilized mercaptopropionic acid (MPA)-capped silver-coated gold nanostars (SGNS@MPA) to enhance the detection capabilities by inducing hydrogen-bonding interactions, which facilitated effective analyte capture (Figure 17). The SGNS@MPA demonstrated a remarkable enrichment ability for creatinine molecules in an alkaline medium (pH-9), leading to nanoparticle aggregation and enhanced SERS signals. The detection limit for creatinine was notably low at 0.1 nM, with a LOD of 14.6 pM. In practical applications, the method achieved detection limits of 0.136 nM and 0.266 nM for saliva and sweat, respectively, without requiring sample separation. Table 3 shows the recent reported SERS-based sensor for creatinine detection. Liu et al. [114] pioneered a new hydrogel diaper structure, providing a novel approach for wearable devices. Unlike other wearable devices that detect sweat, tears, and other bodily fluids, this device format significantly enhances sample collection.

### 4.4. Dopamine

**Figure 18:**
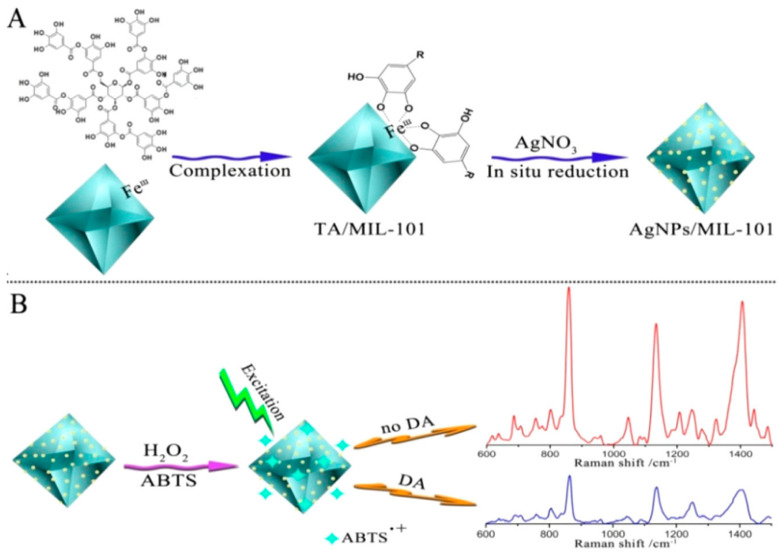
Schematic illustration of the preparation process for the AgNPs/MIL-101 (Fe) hybrid structure (**A**) and schematic diagram of the detection of DA based on SERS (**B**). Reprinted with permission from ref. [122]. Copyright 2015 ACS.

Dopamine, a crucial neurotransmitter, can be found in urine and serves as a biomarker for various neurological and psychiatric disorders. The detection of dopamine using SERS has been significantly enhanced by the development of specialized noble metal nanohybrids [121]. For example, Jiang et al. [122] focused on developing a SERS substrate by synthesizing AgNPs in situ on the surface of a MOF, specifically MIL-101 (Fe). The AgNPs/MIL-101 (Fe) composite was designed to create numerous Raman hot spots due to the high density of AgNPs and the excellent adsorption properties of the MOF (Figure 18). The substrate demonstrated a remarkable detection limit of approximately 0.32 pM for dopamine with a linear detection range from 1.054 pM to 210.8 nM and a correlation coefficient of 0.992. The method also showed high recoveries of 99.8% to 108.0% in human urine samples. Lu et al. [123] developed a SERS-active ZnO/Ag hybrid microcavity utilizing the Whispering Gallery Mode (WGM) effect for the ultrasensitive detection of dopamine. This hybrid microcavity enhanced the Raman signal of rhodamine 6G by over ten times compared to a ZnO film/Ag substrate, demonstrating high sensitivity with a detection limit as low as 10^−12^ M for dopamine. The ZnO/Ag hybrid microcavity, characterized by Ag nanoparticles and WGM-enhanced light-matter interaction, offered significant improvement in sensitivity without complex substrate assembly. The study highlighted the potential of this microcavity for detecting neurotransmitters, benefiting from the natural WGM structure’s ability to confine light waves at the ZnO/air boundary, thus strengthening light-matter interactions. The design showed promise for rapid trace detection in life sciences, supported by a calculated enhancement factor of 1.2 × 10^10^ and a linear response in the Raman intensity for varying concentrations of R6G, underscoring its effectiveness for molecular sensing applications. Miyazaki et al. [124] focused on synthesizing gold-conjugated magnetite nanoparticles (AuMNP) to enhance SERS for detecting dopamine. This approach aimed to address challenges in SERS reproducibility and repeatability due to variables like uncontrolled colloid aggregation and Brownian motion. The AuMNPs were synthesized using a coprecipitation method and characterized by various techniques. When used as a SERS platform, the AuMNPs provided a significant improvement in measurement stability. Under magnetic concentration with a neodymium magnet, the standard deviation of SERS signal intensities was reduced to 19.4 from 800 arbitrary units in dispersion measurements. This method also demonstrated a LOD for dopamine at 10^−6^ M, following the Freundlich isotherm model, indicating multilayer adsorption. Table 4 shows the recent reported SERS-based sensor for dopamine detection.

### 4.5. Other Metabolites and Biomarkers

Beyond the major metabolites discussed above, noble metal nanohybrids have been applied to the SERS detection of various other urinary compounds of clinical significance. These include amino acids, nucleic acid derivatives, and small molecule biomarkers associated with specific diseases or metabolic states.

For instance, a gold nanoparticle–graphene oxide hybrid has been developed for the detection of tryptophan, an essential amino acid. Quezada et al. [132] explored the development of a SERS sensor utilizing MIP nanoparticles (nanoMIPs) for the detection of prostate cancer biomarkers, specifically the amino acids tryptophan and tyrosine. The nanoMIPs were synthesized using a solid-phase molecular imprinting technique and were subsequently immobilized on a SERS substrate with gold nanoparticles. These nanoparticles enhanced the Raman signal, allowing for the sensitive detection of tryptophan and tyrosine in phosphate-buffered saline. The sensor demonstrated impressive detection limits of 7.13 μM for TPP and 22.11 μM for TRS, with quantification limits of 23.75 μM and 73.72 μM, respectively. These values are within the concentration ranges found in patients with prostate cancer, suggesting the sensor’s potential for early diagnosis. The study highlighted the sensor’s specificity through cross-reactivity tests, showing no response to aspartic acid, thereby confirming its selectivity for the target amino acids.

Nucleic acid derivatives, such as guanine and adenine, which can be indicators of oxidative stress and DNA damage, have been detected using colloidal gold clusters [133]. By examining the pH-dependent behavior of these analytes and optimizing the conditions, the study achieved improved repeatability of SERS measurements. The formation of small-sized gold clusters was found to be crucial in enhancing measurement precision. The developed correlation constrained multivariate curve resolution-alternating least squares method effectively resolved overlapping SERS bands, allowing quantification of micromolar concentrations of bioanalytes. This approach provided similar performance to partial least squares regression, with the added advantage of retrieving valuable spectral information.

The detection of bacterial metabolites in urine, which can be indicators of urinary tract infections, has also been explored using noble metal nanohybrids. A paper-based 3D SERS substrate has shown promise in detecting pyocyanin, a metabolite produced by *Pseudomonas aeruginosa* [134]. This method involved a one-pot Au electrodeposition technique that allowed the direct detection of pyocyanin without the need for complex sample preparation. The procedure utilized a hydrogel-coated, AuNPs-decorated filter paper, which enhanced the Raman signal by trapping pyocyanin in the interstitial voids of the growing gold nanostructures. This setup effectively excluded large macromolecules, thus maintaining a clean surface for sensitive detection. The system demonstrated an LOD of 0.56 µM in complex media, which is significantly lower compared to traditional methods.

## 5. Challenges and Future Perspectives

The application of surface-enhanced Raman spectroscopy (SERS) using noble metal nanohybrids for urinary metabolite analysis has shown remarkable progress in recent years. However, several challenges remain to be addressed before this technology can be fully realized in clinical settings. Concurrently, emerging trends and innovative approaches are opening up exciting new avenues for research and development in this field. One of the primary challenges facing SERS-based urinary metabolite analysis is the complex and variable nature of urine samples. The composition of urine can vary significantly between individuals and even within the same individual over time, depending on factors such as diet, hydration status, and overall health. This variability can affect the interaction between metabolites and SERS substrates, potentially leading to inconsistent results. Future research must focus on developing robust calibration methods and standardization protocols that can account for this inherent variability in urine composition.

Another significant challenge lies in achieving high selectivity for specific metabolites in the presence of numerous interfering compounds. While noble metal nanohybrids have shown improved selectivity compared to traditional SERS substrates, further enhancements are needed for reliable quantification of target metabolites in complex urine matrices. The development of hybrid materials with molecular recognition elements, such as aptamers or molecularly imprinted polymers, integrated into their structure represents a promising direction for improving selectivity. The reproducibility of SERS measurements remains a concern, particularly when dealing with trace amounts of metabolites. Variations in hot spot distribution and intensity across the substrate surface can lead to inconsistent signal enhancement. Future efforts should focus on fabrication techniques that produce more uniform and reproducible noble metal nanohybrid substrates. Advanced nanofabrication methods, such as DNA-directed assembly or lithographic techniques, may offer routes to creating highly ordered and reproducible SERS-active structures [135,136].

Translating laboratory-based SERS techniques to point-of-care devices presents another set of challenges. Miniaturization of both the SERS substrate and the detection instrumentation is crucial for developing portable diagnostic tools. The integration of microfluidic systems with SERS-active noble metal nanohybrids offers a promising approach to creating compact [137,138], automated platforms for urinary metabolite analysis. Future research should focus on developing low-cost, disposable SERS chips that can be easily integrated with portable Raman spectrometers. Looking towards the future, several exciting trends are emerging in the field of SERS-based urinary metabolite analysis. The development of multifunctional noble metal nanohybrids that combine SERS enhancement with other modalities, such as fluorescence or electrochemical sensing [139,140], could provide complementary information and improve diagnostic accuracy. These multimodal platforms could offer a more comprehensive analysis of urinary metabolites, potentially uncovering new biomarkers and diagnostic indicators. The miniaturization and portability of Raman lasers require significant time and financial investment from researchers for optimization, and we have high expectations for this development.

In recent years, the rapid development of machine learning models has elevated SERS detection to new heights. One of the key features of machine learning models is their ability to help optimize the interference noise present in Raman spectra, removing interference peaks and preserving the shape of characteristic peaks in each information region of the Raman spectrum [141,142]. Another significant feature is the classification and recognition capabilities of machine learning, which play a crucial role in SERS detection. Machine learning-assisted SERS can achieve multi-target classification and detection by categorizing different targets into distinct sets based on their characteristic peaks, enabling multi-channel detection. This approach can provide a foundation for subsequent clinical detection of multiple target substances in urine samples [143,144]. Machine learning-assisted SERS is particularly valuable in clinical settings because it can predict diseases based on biomarker indicators. This capability contributes to patient prevention and treatment of specific diseases [145,146]. Many efforts have been made towards disease prevention and detection [147]; however, it is often challenging to bring actual patient samples into the laboratory. As a result, much of the work has to rely on simulating the concentrations of disease biomarkers rather than measuring the concentrations in actual patient samples. This is a question that needs to be addressed in the field of big data sensing and clinical detection.

The integration of SERS technology with wearable devices represents another promising avenue for future research [148,149]. The continuous monitoring of urinary metabolites through SERS-enabled smart toilets or wearable sensors could provide real-time health information and early warning signs of various conditions. This approach could revolutionize personalized medicine and preventive healthcare. However, we also need to consider various factors such as aesthetics, comfort, cost, privacy, and security in order to enable SERS wearable devices to be better applied within the general population.

## 6. Conclusions

This comprehensive review has explored the significant advancements in SERS for urinary metabolite analysis, with a particular focus on noble metal nanohybrids. The development of these hybrid materials has dramatically enhanced the sensitivity, selectivity, and reproducibility of SERS-based detection methods for key urinary metabolites such as uric acid, urea, creatinine, and dopamine. Noble metal nanohybrids, including carbon-based, metal–organic-framework (MOF), silicon-based, semiconductor, and polymer-based hybrids, have demonstrated remarkable improvements in SERS performance compared to traditional noble metal nanoparticles. These hybrid materials offer enhanced stability, improved hot spot generation, and the potential for multifunctionality. Notably, detection limits for urinary metabolites have been pushed to unprecedented levels, with some systems achieving detection in the picomolar range. The review has highlighted innovative fabrication techniques, such as in situ growth methods and template-assisted synthesis, which have enabled precise control over nanostructure morphology and composition. Additionally, the integration of SERS with other analytical modalities, such as electrochemistry and microfluidics, has opened new avenues for comprehensive metabolite profiling. Despite these advancements, challenges remain, including the need for improved reproducibility, standardization of measurement protocols, and the development of user-friendly, point-of-care devices. Future directions in this field are likely to focus on the creation of multifunctional sensing platforms, the integration of SERS technology with wearable devices, and the application of advanced data analysis techniques, including machine learning, to extract maximum information from SERS spectra. As research in this area continues to evolve, SERS-based analysis of urinary metabolites using noble metal nanohybrids holds great promise for non-invasive diagnostics, personalized medicine, and early disease detection, potentially revolutionizing healthcare monitoring and clinical diagnostics.

## Figures and Tables

**Table 1:** Summary of recent advancements in SERS-based sensors for uric acid detection.

Material Composition	LOD	Detection Range	Key Features and Applications	Ref.
AgNPs embedded on PDDA modified GO nanosheets.	2.5 × 10^−5^ M	1 × 10^−4^ to 4 × 10^−4^ M	The floating SERS substrate offers rapid, ultra-sensitive, and label-free detection of biomolecules and clinical uremic toxins. It is particularly useful for detecting uremic toxins in blood. The sensor’s design allows it to float in water, facilitating the capture and identification of biomolecules.	[89]
AgNPs embedded on mrGO	10^−6^ M	-	The sensor offers rapid, reproducible, and ultrasensitive detection without labeling. It is particularly effective for detecting uremic toxins and parathyroid hormone, making it suitable for applications in chronic kidney disease diagnostics.	[90]
Au NPs are uniformly modified on CeO_2_ nanorods	3.29 × 10^−10^ M	0.01 to 10,000 μM	It benefits from the excellent catalytic performance of Au/CeO_2_ nanorods. It is used for detecting uric acid in human serum and urine, offering potential applications in biosensing and biomedical diagnostics.	[91]
A core of gold-silver alloy nanoparticles coated with a manganese dioxide shell	7.18 nM	20 nM to 500 μM	The sensor provides dual-mode detection using SERS and electrochemical signals. It is designed for accurate and quantitative detection of uric acid and can also be used for other physiological indicators like glucose, xanthine, choline, and lactate.	[92]
g-C_3_N_4_/AuNPs hybrid, thermally annealed at 350 °C	10^−11^ M	10^−11^ M to 10^−4^ M	Ultrasensitive SERS detection of uric acid in artificial urine. Enhanced photoinduced electron transfer efficiency. Potential for early diagnosis of kidney function and related diseases.	[93]
Au@Ag NPs modified with hexadecyltrimethylammonium chloride	2.5 mg/L	12.5 to 112.5 mg/L	Rapid (30 s) detection of uric acid in urine samples. Uses creatinine as an endogenous internal standard for calibration. No sample pretreatment required besides dilution.	[94]
Gold-coated silver nanostructures with AuNPs	1.16 nM	1 nM to 1 mM	Effective in both serum and urine samples. Potential for real-world clinical applications in detecting uric acid abnormalities.	[95]
Ag_2_S nanowires on fluorine-doped tin oxide glass substrate	0.1 μM	0.2 to 1000 μM	Enzyme-free electrochemical sensor for uric acid detection. Good selectivity against common interferents.	[96]
Multilayered AgNPs on Ti substrate (Ti/AgNFs/AgNPs)	1 μM	-	Uric acid detection in urine samples. Guided design using FDTD simulations.	[97]
AgNPs coated with carbon dots	0.01 μM	0.01 to 500 μM	Synergistic colorimetric and SERS-based detection of uric acid with high sensitivity and selectivity. The nanocomposite exhibits peroxidase-like catalytic activity and SERS enhancement, enabling both visual color change and sensitive SERS signal detection for uric acid sensing.	[98]

**Table 2:** Summary of recent advancements in SERS-based sensors for urea detection.

Material Composition	LOD	Detection Range	Key Features and Applications	Ref.
AuCOF	0.01 nM	0.13 to 3.33 nM	The sensor combines SERS and RRS for dual-mode detection, providing high sensitivity and accuracy. It is used for detecting small organic molecules, with specific applications in biochemical analysis such as detecting urea in biological samples.	[100]
Blu-ray DVDs with AuNPs trapped in nanochannels.	0.084 μg/mL	-	This sensor was designed for cost-effective and reliable detection of clinically important chemicals in urine, particularly for medical diagnostics related to kidney function by detecting albumin, creatinine, and urea. The substrate demonstrated stability over 45 days.	[101]
AuNPs on untreated aluminum foil	24 mM	0.03 to 0.15 M	The sensor is cost-effective, easy to prepare, and suitable for detecting biomarkers like urea in urine. It provides reliable quantification within clinically relevant ranges.	[102]
AgNPs functionalized with 4-mercaptopyridine and stabilized with BSA, encapsulated in an alginate hydrogel	0.1 mM	0.1 mM to 10 mM	High sensitivity and stability due to BSA preventing nanoparticle aggregation. The encapsulation method allows for versatility in targeting different analytes by altering the enzyme used.	[103]
Laser-induced graphene (LIG) substrates with electrochemically deposited AuNPs	10^−7^ M	10^−2^ M to 10^−6^ M	The sensor combines SERS and electrochemistry (EC) for enhanced sensitivity and specificity. It is suitable for point-of-care diagnostics and remote monitoring of kidney function.	[104]
Plasmonic cellulose microfilament coated with AgNPs	10^−5^ M	-	It is particularly useful for online detection in microfluidics. The integration with deep learning enhances its diagnostic accuracy, making it suitable for trace substance identification.	[105]
Periodic gold nanodisk arrays on glass substrates	0.01 mM	0.05 mM to 10 mM	High enhancement factor of 2.3 × 10^6^ for the 300-nm period substrate, making it suitable for practical SERS applications.	[106]
Nanoporous gold disk arrays integrated into a microfluidic channel	0.67 mM	-	Rapid detection (<2 s), label-free sensing, high reproducibility, and versatility for detecting neurotransmitters and metabolites.	[107]
Raspberry-shaped Au nanostructures on Si wafer substrate	4.17 μM	10 to 500 mM	Flexible wearable diaper sensor for label-free, multiplex detection of urea, creatinine and bilirubin in urine using SERS. Enables non-invasive urinalysis and potential early disease screening using a handheld Raman spectrometer.	[108]
Dendritic silver microparticles on ITO glass substrate	0.15 mg/mL	0.2–10 mg/mL	Potential applications in clinical analysis for monitoring kidney function and in food processing technology. The dendritic silver structure provides strong SERS enhancement, allowing for detection within the physiological range of urea concentrations.	[109]

**Table 3:** Summary of recent advancements in SERS-based sensors for creatinine detection.

Material Composition	LOD	Detection Range	Key Features and Applications	Ref.
A composite of AuNPs embedded within a MIL-101 (Fe)	0.1 μM	1 μM to 100 μM	The method benefits from the electrostatic interaction between the MOF and creatinine, enhancing detection sensitivity.	[111]
h-BN/Au NCs with a controllable gold composition, optimized at 1.68%	-	10⁻^2^ to 10⁻⁶ M	The SERS activity was enhanced by mechanisms such as electromagnetic and charge-transfer enhancements.	[112]
MPA-capped SGNS@MPA	14.6 pM	0.1 nM to 500 μM	The sensor demonstrated high sensitivity and specificity for creatinine detection in human fluids like saliva and sweat without requiring sample separation. It is particularly suitable for point-of-care diagnostics and applications.	[113]
Silver nanowires and silver nanocubes embedded in a gelatin/polyvinyl alcohol hydrogel	0.59 μM	10^−2^ M to 10^−6^ M	Wearable diaper-based SERS sensor for simultaneous detection of multiple biomarkers (creatinine, uric acid, bilirubin) and pH in urine. Offers high sensitivity, good stability, antimicrobial properties, and biocompatibility. Suitable for non-invasive, real-time monitoring of metabolites and pH in urine for health assessment.	[114]
Polyelectrolyte multilayers over a gold film, utilizing polyacrylic acid and polyallylamine hydrochloride for the layer-by-layer self-assembly process	1.47 µM	1 µM to 10 mM	This sensor is designed for selective and label-free detection of creatinine. It shows high sensitivity, selectivity, and uniformity, making it suitable for clinical diagnostics, particularly in assessing renal function through creatinine detection in urine and serum.	[115]
Nanoporous copper and silver nanoflowers created through chemical etching and galvanic displacement	1.7 pg/mL	-	The use of low-cost and readily available materials like brass makes it an economical choice for developing high-quality SERS substrates.	[116]
AuNPs and cucurbit[7]uril	12.5 ng/mL	0.06 to 1.50 μg/mL	Dual detection via SERS and UV-Vis spectroscopy. Highly sensitive and reproducible detection in synthetic urine. Tolerant to matrix effects and protein fouling.	[117]
3D hybrid substrate with two layers—a bottom layer of Au@MGITC@SiO_2_ nanoparticles as the calibration unit, and a top layer of Au octahedrons as the detection unit.	2 mg/L	0.1 to 100 mg/L	The sensor provides self-calibration ability through the built-in internal standard, avoiding competitive adsorption issues. It demonstrates improved SERS performance and reproducibility compared to 2D substrates.	[118]
Silver dendritic nanostructures on gold microelectrodes	0.11 mg/L	2 to 320 mg/L	Label-free, electric field-assisted SERS sensor. Detects creatinine and HSA in artificial urine. Uses dielectrophoresis and electrothermal fluid flow to concentrate analytes. Forms a sandwich assay with silver nanoparticles to amplify SERS signal.	[119]
Nanostructured polychloro-p-xylylene film coated with a thin layer (~60 nm) of AgNPs	0.5 μg/mL	0.5 to 10.2 μg/mL	Provides fast (~10 s) detection with low laser power. Resistant to differences in ionic strength. Applicable for early detection of kidney diseases and monitoring renal function in diabetic patients.	[120]

**Table 4:** Summary of recent advancements in SERS-based sensors for dopamine detection.

Material Composition	LOD	Detection Range	Key Features and Applications	Ref.
AgNPs synthesized in situ on the surface of the MIL-101 (Fe) using tannic acid	0.32 pM	1.054 pM to 210.8 nM	The sensor features high sensitivity due to numerous Raman hot spots and excellent adsorption performance. It is used for ultrasensitive detection of dopamine and shows potential for chemical and biological assay applications.	[122]
ZnO/Ag hybrid microcavity with AgNPs	10^−12^ M	-	High SERS sensitivity due to WGM and LSPR effects, potential for detecting neurotransmitters like dopamine in life sciences.	[123]
AuMNP	10^−6^ M	-	The sensor improves SERS signal reproducibility and repeatability by using magnetic concentration, which reduces the standard deviation of signal intensities significantly. It is used for detecting dopamine, leveraging multilayer adsorption and dual hot spots for enhanced performance.	[124]
An Au nanopillar electrode modified by in situ electrochemical deposition of Au nanostructures	0.1 nM	0.1 nM to 0.1 mM	This sensor is notable for its high sensitivity and selectivity in the simultaneous label-free detection of ascorbic acid, uric acid, and dopamine in complex biological fluids like urine.	[125]
silver nanocubes and a nanoporous silver film functionalized with mercaptopropionic acid and 4-mercaptobenzene boronic acid	40 fM	10^−4^ M to 10^−13^ M	It is particularly useful for detecting dopamine in complex biological fluids like serum and urine, with potential applications in the early diagnosis of diseases related to abnormal dopamine levels, such as Parkinson’s disease.	[126]
AgNPs decorated on graphene oxide nanoribbons	0.67 nM	5 to 2500 nM	Label-free SERS detection of dopamine using rhodamine 6G as a Raman reporter. The sensor shows potential for dopamine detection in biomedical applications and fundamental studies in science and medicine.	[127]
AgNPs coated with polyvinylpyrrolidone	40 nM	0.1 to 10 μM	This sensor uses a nanozyme catalyzed SERS technique for dopamine detection. It employs AgNPs@PVP with oxidase-like activity to catalyze TMB oxidation without hydrogen peroxide.	[128]
rGO/silver nanotriangle composite sol with acridine red as a label-free molecular probe	1.2 μM	2.5 to 500 μM	The sensor utilizes the strong affinity of dopamine to rGO to displace acridine red molecules, which then adsorb on AgNT aggregates to produce a strong SERS signal proportional to dopamine concentration.	[129]
AuNPs immobilized on thiol-functionalized PEDOT films	37 nM	50 nM to 100 μM	Hybrid organic electrochemical transistor and SERS sensor. Selective detection of dopamine in presence of interferents. SERS-based detection of p-cresol.	[130]
AgNPs modified with iron–nitrilotriacetic acid as the dopamine-selective probe, and 4-mercaptobenzoic acid as an internal standard.	0.04 μM	0.12 μM to 3.5 μM	Can quantify dopamine in complex biological samples like plasma and urine. Uses a multiplicative effects model (MEM-SERS) for improved quantitative accuracy.	[131]

## Data Availability

Data are contained within the article.
